# Supplementary material for: Integrating multi-type aberrations from DNA and RNA through dynamic mapping gene space for subtype-specific breast cancer driver discovery
Source: PeerJ. 2023 Feb 3;11:e14843. doi: 10.7717/peerj.14843 (PMC9901305; doi:10.7717/peerj.14843)
Supplement: Supplemental Information 1 [file peerj-11-14843-s001.pdf]

Supplementary Table 1. The full list of the top ranked candidates of our identified results, which contains considerable numbers of novel drivers.

Rank	Gene	Benchmark	InOthers	Rank	Gene	Benchmark	InOthers	Rank	Gene	Benchmark	InOthers	Rank	Gene	Benchmark	InOthers	Rank	Gene	Benchmark	InOthers
1	AKT1	Known	Yes	2	EP300	Known	Yes	3	IGH	Known	No	4	C15orf65	Known	No	5	C15orf65	Unknown	Yes
6	TNFRSF17	Known	No	7	DAXX	Known	No	8	ESR1	Known	Yes	9	SMAD3	Known	Yes	10	NCOR2	Known	Yes
11	PHOX2B	Known	No	12	PWWP2A	Known	Yes	13	JAZF1	Known	Yes	14	BRCA1	Known	Yes	15	CRLF2	Known	No
16	BCL5	Known	No	17	STAT3	Known	Yes	18	HOXA11	Known	Yes	19	HMGN2P46	Known	No	20	RMI2	Known	No
21	C12orf9	Known	No	22	AR	Known	Yes	23	IGK	Known	Yes	24	CREBBP	Known	Yes	25	SLC45A3	Known	Yes
26	TAL2	Known	No	27	LHFP	Known	No	28	MLLT11	Known	No	29	BCR	Known	No	30	RARA	Known	Yes
31	SPOP	Known	No	32	RNF217-AS1	Known	Yes	33	CNOT3	Known	Yes	34	IL2	Known	No	35	ARNTL	Unknown	No
36	SMAD4	Known	No	37	CD274	Known	Yes	38	BCL5	Unknown	No	39	MTCP1	Known	Yes	40	CDC73	Known	No
41	CDC73	Unknown	Yes	42	ATG12	Unknown	No	43	CIITA	Known	Yes	44	CIITA	Unknown	No	45	AKT1	Unknown	No
46	DDX10	Known	Yes	47	DAXX	Unknown	No	48	FHIT	Known	No	49	TTL	Known	No	50	CTNNB1	Unknown	Yes
51	CTNNB1	Unknown	No	52	GATA1	Known	No	53	COX6C	Known	Yes	54	NUTM1	Known	Yes	55	CCNB1	Unknown	Yes
56	TP53	Known	Yes	57	PIM1	Known	No	58	NFIB	Known	Yes	59	RB1	Known	Yes	60	SOX2	Known	No
61	MALAT1	Known	No	62	NCOA1	Known	Yes	63	EGFR	Known	Yes	64	BRCA1	Unknown	Yes	65	PTK6	Known	Yes
66	BCL7A	Known	Yes	67	CHIC2	Known	Yes	68	SMARCA4	Known	Yes	69	ASXL1	Known	Yes	70	BTG1	Known	Yes
71	RUNX1	Known	Yes	72	C12orf9	Unknown	No	73	DROSHA	Known	Yes	74	CREBBP	Unknown	No	75	MSH6	Known	Yes
76	BCL7A	Unknown	Yes	77	CD79B	Known	Yes	78	PHF6	Known	No	79	IGL	Known	Yes	80	PDGFRB	Known	Yes
81	CIC	Known	No	82	BCL11B	Known	No	83	CDKN2A	Known	No	84	CDX2	Known	No	85	CREB1	Known	No
86	SSX1	Known	No	87	PBRM1	Known	Yes	88	ETV4	Known	No	89	H3F3B	Known	Yes	90	CLTC	Known	Yes
91	CCND1	Known	No	92	CCND1	Unknown	Yes	93	BTRC	Unknown	Yes	94	CEP89	Known	Yes	95	LRP1B	Known	No
96	PAX7	Known	Yes	97	CHIC2	Unknown	Yes	98	AMER1	Known	Yes	99	AHR	Unknown	No	100	EBF1	Known	Yes
101	PLAG1	Known	No	102	STAT5B	Known	Yes	103	DDR2	Known	Yes	104	DDX3X	Known	Yes	105	SMARCB1	Known	Yes
106	MALT1	Known	Yes	107	LCK	Known	Yes	108	RSPO2	Known	Yes	109	LMO2	Known	Yes	110	CAMTA1	Known	No
111	APC	Known	Yes	112	XPO1	Known	Yes	113	CTCF	Known	No	114	BTG1	Unknown	Yes	115	MYCN	Known	Yes
116	CRTC1	Known	No	117	AKR1C1	Unknown	Yes	118	ETV6	Known	Yes	119	POU2AF1	Known	No	120	DNM2	Known	Yes
121	GATA2	Known	Yes	122	CDC25A	Unknown	Yes	123	EGFR	Unknown	Yes	124	HLF	Known	Yes	125	RANBP2	Known	Yes
126	CCNB1IP1	Known	No	127	TRD	Known	No	128	TFRC	Known	Yes	129	SF3B1	Known	Yes	130	TAF15	Known	Yes
131	ELK4	Known	No	132	CD79A	Known	No	133	BCL11A	Known	No	134	WIF1	Known	No	135	P2RY8	Known	No
136	AKT2	Known	Yes	137	ABL2	Known	Yes	138	MAPK1	Known	No	139	H3F3A	Known	No	140	ETV1	Known	No
141	ALDH2	Known	Yes	142	YWHAE	Known	Yes	143	SMAD2	Known	Yes	144	PIK3R1	Known	Yes	145	TCF3	Known	Yes
146	HSP90AA1	Known	Yes	147	ECT2L	Known	No	148	FSTL3	Known	Yes	149	CALCOCO1	Unknown	No	150	ENSG00000119397	Unknown	No
151	CAMTA1	Unknown	No	152	CHD4	Known	Yes	153	MYB	Known	Yes	154	ASXL1	Unknown	No	155	CDKN1B	Known	Yes
156	NPM1	Known	No	157	MET	Known	Yes	158	HNF1A	Known	Yes	159	PAX5	Known	No	160	HMGA2	Known	No
161	CREB3L2	Known	No	162	FGFR4	Known	Yes	163	CHCHD7	Known	No	164	PDGFRA	Known	Yes	165	CCNE1	Known	No
166	TCF12	Known	Yes	167	EML4	Known	No	168	EML4	Unknown	No	169	TGFBR2	Known	Yes	170	MNX1	Known	No
171	PTEN	Known	Yes	172	CHUK	Unknown	Yes	173	FAS	Known	No	174	DYNLL2	Unknown	No	175	PBX1	Known	Yes
176	JUN	Known	Yes	177	CDKN2C	Known	Yes	178	CDKN2C	Unknown	No	179	NCOR1	Known	Yes	180	IDH2	Known	Yes
181	HOXC11	Known	No	182	HSP90AB1	Known	Yes	183	ERBB2	Known	Yes	184	CBLB	Known	Yes	185	CBLB	Unknown	No
186	FLI1	Known	Yes	187	KCNJ5	Known	No	188	FAM46C	Known	Yes	189	SET	Known	Yes	190	STK11	Known	Yes
191	MAP3K13	Known	Yes	192	NTRK1	Known	No	193	MLLT1	Known	No	194	CREB3L1	Known	Yes	195	ASPSCR1	Known	Yes
196	ASPSCR1	Unknown	No	197	MDM2	Known	Yes	198	DDB2	Known	No	199	CRTC1	Unknown	Yes	200	CCDC6	Known	No
201	FEV	Known	Yes	202	CEP89	Unknown	No	203	SFPQ	Known	No	204	DUX4L1	Known	No	205	DUX4L1	Unknown	No
206	DHX9	Unknown	Yes	207	CARS	Known	No	208	DDR2	Unknown	No	209	CARD11	Known	No	210	TLX1	Known	Yes
211	CRK	Unknown	Yes	212	CHD4	Unknown	Yes	213	PLCG1	Known	Yes	214	NT5C2	Known	No	215	HOXD11	Known	No
216	AR	Unknown	No	217	DNMT3A	Known	No	218	RBM15	Known	No	219	IDH1	Known	No	220	IL21R	Known	Yes
221	HIST1H4I	Known	No	222	HIF1A	Known	Yes	223	EZR	Known	Yes	224	FGFR3	Known	Yes	225	CASP6	Unknown	Yes
226	RANBP17	Known	No	227	MLLT4	Known	Yes	228	NAB2	Known	Yes	229	LIFR	Known	Yes	230	COL8A1	Unknown	No
231	SS18L1	Known	Yes	232	PCSK7	Known	No	233	CDX2	Unknown	No	234	CDH1	Known	Yes	235	ETNK1	Known	Yes
236	RUNX1T1	Known	Yes	237	ARHGDIA	Unknown	Yes	238	PICALM	Known	Yes	239	KRAS	Known	No	240	BCR	Unknown	No
241	IKZF1	Known	Yes	242	CUX1	Known	Yes	243	CARM1	Unknown	Yes	244	TFPT	Known	No	245	LYL1	Known	No
246	BCL3	Known	No	247	KIAA1598	Known	No	248	DEK	Known	No	249	DNM1	Unknown	Yes	250	BCORL1	Known	No
251	ATG3	Unknown	No	252	ERG	Known	Yes	253	CCNB1IP1	Unknown	No	254	EPS15	Known	No	255	PIK3CA	Known	Yes
256	TCEA1	Known	Yes	257	BARD1	Unknown	Yes	258	BCL2	Known	No	259	RAD21	Known	Yes	260	CD5	Unknown	No
261	SH3GL1	Known	No	262	ELP3	Unknown	No	263	DDX24	Unknown	No	264	CFL1	Unknown	No	265	AMER1	Unknown	No
266	EEF1A1	Unknown	No	267	TFEB	Known	Yes	268	DISC1	Unknown	Yes	269	ZNF384	Known	No	270	ERC1	Known	No
271	CASP1	Unknown	No	272	GATA3	Known	Yes	273	BCL9	Known	No	274	ATRX	Known	No	275	ELL	Known	Yes
276	ELL	Unknown	No	277	ATF7IP	Unknown	Yes	278	HNRNPA2B1	Known	No	279	NUP98	Known	No	280	CCND3	Known	Yes
281	CCND3	Unknown	No	282	CREB3L1	Unknown	Yes	283	RHOH	Known	Yes	284	ARHGAP26	Known	No	285	APC	Unknown	No
286	JAK1	Known	Yes	287	ATG16L2	Unknown	Yes	288	CSF3R	Known	No	289	GNAS	Known	Yes	290	EED	Unknown	No
291	MSI2	Known	No	292	CHAF1A	Unknown	Yes	293	WT1	Known	No	294	BCL3	Unknown	Yes	295	CBFA2T2	Unknown	Yes
296	BCOR	Known	No	297	DDX5	Known	Yes	298	DDX5	Unknown	No	299	ATP1A1	Known	Yes	300	ATP1A1	Unknown	No
301	CLTCL1	Known	Yes	302	KIAA1549	Known	Yes	303	GPHN	Known	Yes	304	CTBP1	Unknown	Yes	305	HOXC13	Known	Yes
306	RBM10	Known	Yes	307	NUP214	Known	Yes	308	ALG13	Unknown	Yes	309	EIF3E	Known	No	310	C3	Unknown	Yes
311	MYC	Known	Yes	312	CHCHD7	Unknown	No	313	DDIT3	Known	No	314	CDK1	Unknown	No	315	DNMT3A	Unknown	Yes
316	MEN1	Known	Yes	317	SDC4	Known	No	318	ELN	Known	Yes	319	RNF213	Known	No	320	CCNA2	Unknown	No
321	CSNK1A1	Unknown	No	322	NFKB2	Known	Yes	323	AP2S1	Unknown	Yes	324	CREB1	Unknown	No	325	MITF	Known	No
326	PTPRC	Known	No	327	FGFR2	Known	Yes	328	ARHGAP26	Unknown	No	329	BCL10	Known	No	330	ERBB3	Known	Yes
331	BRD3	Known	No	332	BRD3	Unknown	Yes	333	CCT7	Unknown	No	334	LZTR1	Known	No	335	BIRC3	Known	No
336	DDX3X	Unknown	Yes	337	BCORL1	Unknown	No	338	PCM1	Known	Yes	339	TPR	Known	Yes	340	FBXW7	Known	Yes
341	ENSG00000092199	Unknown	No	342	BCL6	Known	No	343	ATP2B3	Known	Yes	344	CSNK2A1	Unknown	Yes	345	STRN	Known	Yes
346	TRIM33	Known	No	347	CANT1	Known	No	348	CANT1	Unknown	No	349	CLIP1	Known	No	350	MTOR	Known	Yes
351	SSX2	Known	No	352	EWSR1	Known	Yes	353	PTPN13	Known	No	354	STAG2	Known	Yes	355	EHMT1	Unknown	No
356	DCC	Unknown	Yes	357	FCGR2B	Known	No	358	LMO1	Known	Yes	359	ACVR1	Known	No	360	ACVR1	Unknown	No
361	COL2A1	Known	Yes	362	EHMT2	Unknown	No	363	THRAP3	Known	No	364	RPL22	Known	No	365	ATXN1	Unknown	Yes
366	DDX10	Unknown	No	367	TAL1	Known	No	368	CBL	Known	Yes	369	DRD4	Unknown	No	370	PTPRB	Known	Yes
371	PPFIBP1	Known	No	372	TBL1XR1	Known	Yes	373	DICER1	Known	No	374	REL	Known	Yes	375	SEPT6	Known	No
376	CREB3L2	Unknown	No	377	ALK	Known	Yes	378	SETBP1	Known	No	379	TCL6	Known	No	380	ATM	Known	Yes
381	CFD1	Unknown	Yes	382	25937	Unknown	No	383	BCL6	Unknown	No	384	FNBP1	Known	No	385	LEF1	Known	No
386	MKL1	Known	Yes	387	CALM3	Unknown	Yes	388	USP6	Known	No	389	FAM131B	Known	No	390	DDX18	Unknown	Yes
391	SND1	Known	Yes	392	ELK1	Unknown	No	393	AXIN2	Known	No	394	VTI1A	Known	Yes	395	CBFB	Known	Yes
396	KMT2D	Known	Yes	397	ANP32A	Unknown	No	398	VHL	Known	No	399	CBFB	Unknown	No	400	AXIN1	Known	Yes
401	MAX	Known	Yes	402	BSG	Unknown	No	403	DNAJA3	Unknown	No	404	MAFB	Known	Yes	405	CALR	Known	No
406	CBLC	Known	Yes	407	CBLC	Unknown	No	408	CTLA4	Unknown	Yes	409	KIF5B	Known	No	410	CCNA1	Unknown	Yes
411	SOCS1	Known	No	412	CTNND2	Unknown	No	413	FLT4	Known	No	414	RAC1	Known	Yes	415	EIF3B	Unknown	Yes
416	TERT	Known	No	417	COL1A1	Known	Yes	418	CCL7	Unknown	No	419	NONO	Known	No	420	KDM5A	Known	Yes
421	CDH2	Unknown	Yes	422	CELSR2	Unknown	No	423	FOXA1	Known	Yes	424	DHX35	Unknown	Yes	425	CD79A	Unknown	No
426	CYLD	Known	No	427	RALGDS	Known	No	428	NR4A3	Known	No	429	DDX1	Unknown	No	430	ATN1	Unknown	Yes
431	LASP1	Known	No	432	CASP8	Known	Yes	433	BCL2	Unknown	No	434	ASCC2	Unknown	Yes	435	ATP2B3	Unknown	Yes
436	PAX3	Known	No	437	KDM5C	Known	No	438	CDK12	Known	No	439	AIRE	Unknown	Yes	440	ZFHX3	Known	No
441	TCF7L2	Known	Yes	442	ERBB4	Known	Yes	443	BORA	Unknown	Yes	444	CD74	Known	No	445	MYH9	Known	Yes
446	CENPJ	Unknown	Yes	447	GNA11	Known	Yes	448	RET	Known	No	449	CAPZB	Unknown	No	450	FUS	Known	No
451	NDRG1	Known	Yes	452	AP2M1	Unknown	Yes	453	ELK4	Unknown	No	454	ATXN2L	Unknown	Yes	455	BRCA2	Known	Yes
456	NCKIPSD	Known	Yes	457	CACNA1D	Known	No	458	STAT6	Known	No	459	SUFU	Known	No	460	FOXL2	Known	Yes
461	TET2	Known	No	462	KDM6A	Known	No	463	KIT	Known	Yes	464	DDIT3	Unknown	Yes	465	PDCD1LG2	Known	No
466	WHSC1L1	Known	Yes	467	NF2	Known	No	468	CNBP	Known	Yes	469	CUEDC2	Unknown	No	470	GAS7	Known	Yes
471	CSK	Unknown	No	472	ATF4	Unknown	Yes	473	CCL2	Unknown	Yes	474	IRF4	Known	No	475	DLG3	Unknown	No
476	GRIN2A	Known	Yes	477	BRCA2	Unknown	No	478	ETV5	Known	No	479	EBF1	Unknown	Yes	480	E2F1	Unknown	Yes
481	BAZ1B	Unknown	Yes	482	COPS6	Unknown	Yes	483	CYLD	Unknown	Yes	484	TSC1	Unknown	Yes	485	LSM14A	Unknown	No
486	FLT3	Known	Yes	487	NOTCH1	Known	No	488	ADAM15	Unknown	Yes	489	CSF3R	Unknown	No	490	MLF1	Unknown	No
491	CBFA2T3	Known	No	492	CCNE1	Unknown	No	493	DNAJB1	Known	No	494	TRIM24	Known	No	495	DICER1	Unknown	No
496	Bub1A	Unknown	No	497	KTN1	Known	Yes	498	NRAS	Known	Yes	499	CUL7	Unknown	Yes	500	DNAJB1	Unknown	Yes
