# Supplementary material for: Integrating multi-type aberrations from DNA and RNA through dynamic mapping gene space for subtype-specific breast cancer driver discovery
Source: PeerJ. 2023 Feb 3;11:e14843. doi: 10.7717/peerj.14843 (PMC9901305; doi:10.7717/peerj.14843)
Supplement: Supplemental Information 2 — (A) and (B) show the front view of the 3D visualization results with interaction source of STRING and iRefIndex, respectively. (C) and (D) show the top view of the 3D visualization results with interaction source of STRING and iRefIndex, respectively. (E) and (F) show the side view of the 3D visualization results with interaction source of STRING and iRefIndex, respectively. [file peerj-11-14843-s002.pdf]

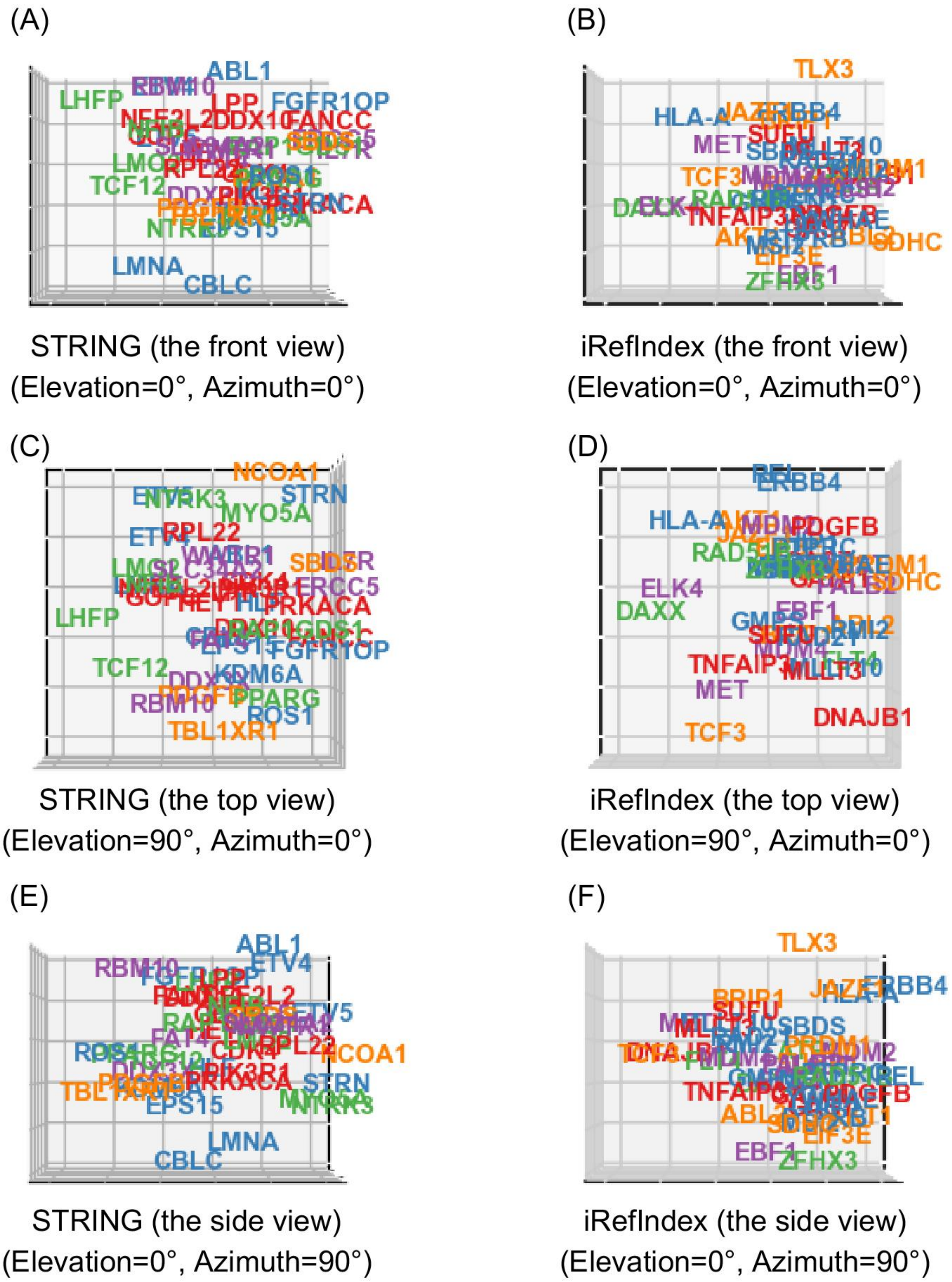

Supplementary Figure 1. The three view drawings of the t-SNE 3D visualization for subtype-specificities of discovered drivers by our approach, including the front view, the top view, and the side view of the 3D visualization. (A) and (B) show the front view of the 3D visualization results with interaction source of STRING and iRefIndex, respectively. (C) and (D) show the top view of the 3D visualization results with interaction source of STRING and iRefIndex, respectively. (E) and (F) show the side view of the 3D visualization results with interaction source of STRING and iRefIndex, respectively.
